# Does the Dual Mobility Cup Reduce Dislocation After Primary Total Hip Arthroplasty in Elderly Patients at High Risk of Dislocation?

**DOI:** 10.1111/os.13613

**Published:** 2022-12-07

**Authors:** Mingliang Chen, Eiji Takahashi, Ayumi Kaneuji, Yoshiyuki Tachi, Makoto Fukui, Yugo Orita, Toru Ichiseki, You Zhou, Norio Kawahara

**Affiliations:** ^1^ Department of Orthopaedic Surgery Kanazawa Medical University Uchinada Japan; ^2^ Department of Orthopaedics Affiliated Renhe Hospital of China Three Gorges University Yichang China

**Keywords:** Dislocation, Dual mobility cup, Pelvic tilt, Supine position, Total hip arthroplasty

## Abstract

**Objective:**

The dual mobility cup (DMC) is designed to extend the longevity of the prosthesis by improving stability, enhancing the range of motion, and decreasing impingement without increasing wear. We hypothesized that DMC would reduce the risk of dislocation in elderly patients. This study aimed to investigate the clinical and radiographic outcomes of DMC‐total hip arthroplasty (THA) in elderly patients at high risk of dislocation.

**Methods:**

From June 2016 to March 2020, 94 patients with a mean age of 77.7 years (97 hips) who underwent a posterolateral approach for DMC‐THA in our department were followed up for at least one year. Preoperative and postoperative pelvic tilt angles (PTA) and DMC orientation were prospectively collected for all patients. Intraoperative and postoperative complications were recorded. A parametric test was used for normal distribution, and a non‐parametric test was used for non‐normal distribution.

**Results:**

Abduction and anteversion angles of the cup were 42.4 and 18.0° in the supine position immediately postoperative. The average PTA for patients in the supine and standing positions were 26.5 and 34.5°, respectively. When moving from the supine to the standing position, patients experienced a mean posterior pelvic tilt of 9°. No intraoperative acetabular‐related complications were recorded. Postoperative complications included early infection in one patient (1.0%) and dislocation in one patient (1.0%).

**Conclusion:**

Our study demonstrates that DMC‐THA provides satisfactory short‐term outcomes in elderly patients at a high risk of dislocation, regardless of the change in PTA resulting from postural transition.

## Graphical abstract

We hypothesized that dual mobility cup (DMC) would help elderly patients avoid dislocation. Additionally, we analyzed the clinical and radiographic outcomes of DMC‐total hip arthroplasty (THA) in elderly patients at high risk of dislocation due to pelvic tilt. The results indicate that DMC‐THA has satisfactory short‐term outcomes in elderly patients at high risk of dislocation, regardless of the change in pelvic tilt angle caused by the patient's position change.

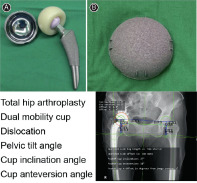

## Introduction

In recent decades, the average age of patients undergoing total hip arthroplasty (THA) has increased worldwide. In the United States, dislocation remains the leading cause of THA revision, with about 17.3% of revision cases due to postoperative dislocation.^1^ The incidence of postoperative dislocation after THA has been reported to be approximately 1%, 2%, and 7% within one month, the first year, and 25 years, respectively.^2^ Gausden *et al*. found that 1.4% of patients (2842/207,285) had a post‐THA median of 40 days due to dislocation‐related readmissions.^3^

The relationship between the spine, pelvis and hip joints has become a topic of considerable interest in recent years, leading to the concept of hip‐spine syndrome.^4^ Spine‐pelvis mobility is age‐related, and kyphosis increases with age.^5^ To maintain sagittal balance, the pelvis compensates by tilting posteriorly. This change increases the risk of posterior acetabular impingement and anterior dislocation in the standing position in patients with THA.^6^ In addition, the pelvis tilts during postural transitions from supine to sitting and standing.^7^ The association between the change in pelvic tilt angles (PTA) and the acetabular anteversion angle has been confirmed to be 1:0.7 to 1:1.^8^, ^9^ When the postoperative change in the posterior pelvic tilt is greater than 20°, the risk of excessive limb loading and posterior impingement increases significantly.^10^ This change can lead to increased mobility and wear for the hip prosthesis and even hip dislocation. Because older age is a risk factor for such dislocation, attention must be focused on preventing dislocation in elderly patients after primary THA.

The dual mobility cup (DMC) has been reported to have advantages such as reducing the risk of dislocation after THA and providing greater mobility.^11^ By treating 97 patients with displaced femoral neck fractures with a mean age of 76.6 years with the DMC, Sunilkumar *et al*. found a reoperation rate of 4.1% and that DMC was able to reduce the THA postoperative risk of early dislocation.^12^

To our knowledge, no study reports the relationship between postoperative pelvic tilt and the risk of dislocation in elderly patients with DMC‐THA. The purpose of this study was: (i) to understand the clinical outcomes and related complications after DMC‐THA in elderly patients; (ii) to understand the early radiographic outcomes after DMC‐THA; and (iii) to investigate the pelvic parameters and prosthesis orientation to assess the role of DMC in THA in elderly patients.

## Patients and Methods

### Study Subjects and Demographic Characteristics

From June 2016 to March 2020, our department performed THA using DMC in 94 patients (97 hips). Inclusion criteria: (i) age ≥70 years; (ii) primary THA; and (iii) definite clinical and imaging diagnosis and required THA. Exclusion criteria: (i) revision cases; an d(ii) less than one year of follow‐up. The group included eight patients (8.5%) with dementia and one (1.1%) with Parkinson's disease. Nearly all participants (89 patients, 91.8%) had risk factors for postoperative dislocation, including developmental dysplasia of the hip (DDH), femoral neck fracture, rapidly destructive coxarthrosis (RDC), proximal femur fracture after internal fixation, subchondral insufficiency fracture (SIF) of the femoral head, and rheumatoid hip osteoarthritis. The demographic data and primary etiologies of the study population are shown in Table 1. The study was approved by the Ethics Review Committee of Kanazawa Medical University (No.134).

**TABLE 1:** Baseline characteristics of the study cohort

Parameter	Values
Sex (male/female)	11/83
Age (years) (range)	77.7 (70–92)
Follow‐up time (months)	22.2 (11.5–50)
Etiology	
Developmental dysplasia of the hip (DDH) (I/II/III/IV)	58 hips (38/10/9/1)
Degenerative arthritis of the hip (primary OA)	8 hips
Femoral neck fracture	12 hips
Rapidly destructive coxarthrosis (RDC)	11 hips
Postoperative internal fixation of proximal femur fracture	3 hips
Subchondral insufficiency fracture of the femoral head (SIF)	4 hips
Rheumatoid arthritis of the hip	1 hip

### Surgical Techniques

A posterolateral approach without a navigation system was used in all patients, and we routinely preserved the pyriform muscle. An acetabular cup was fixed using the “line‐to‐line” technique with screw fixation. The targeted acetabular abduction and anteversion angle of the cup is 40 degrees and 20 degrees in postoperative supine X‐ray images. Regarding pelvic movement, if the patients had posterior pelvic tilting in the standing position, the target for cup anteversion was less than 20 degrees. The Dual Mobility G7 System (Zimmer‐Biomet, Warsaw, IN, USA) was used in all patients. In confirming the stem anteversion angle, we rotated the patient's lower leg internally until it was perpendicular to the ground, which facilitated the determination of the stem inversion angle. We usually use a 30° stem anteversion angle. After the prosthesis was fixed, we repaired the short muscles that controlled external rotation and reconstructed the joint capsule. We generally selected cemented femoral stems for patients with osteoporosis (37 hips) and cementless stems for the others (60 hips).

### Radiographic Analysis

**Fig. 1:**
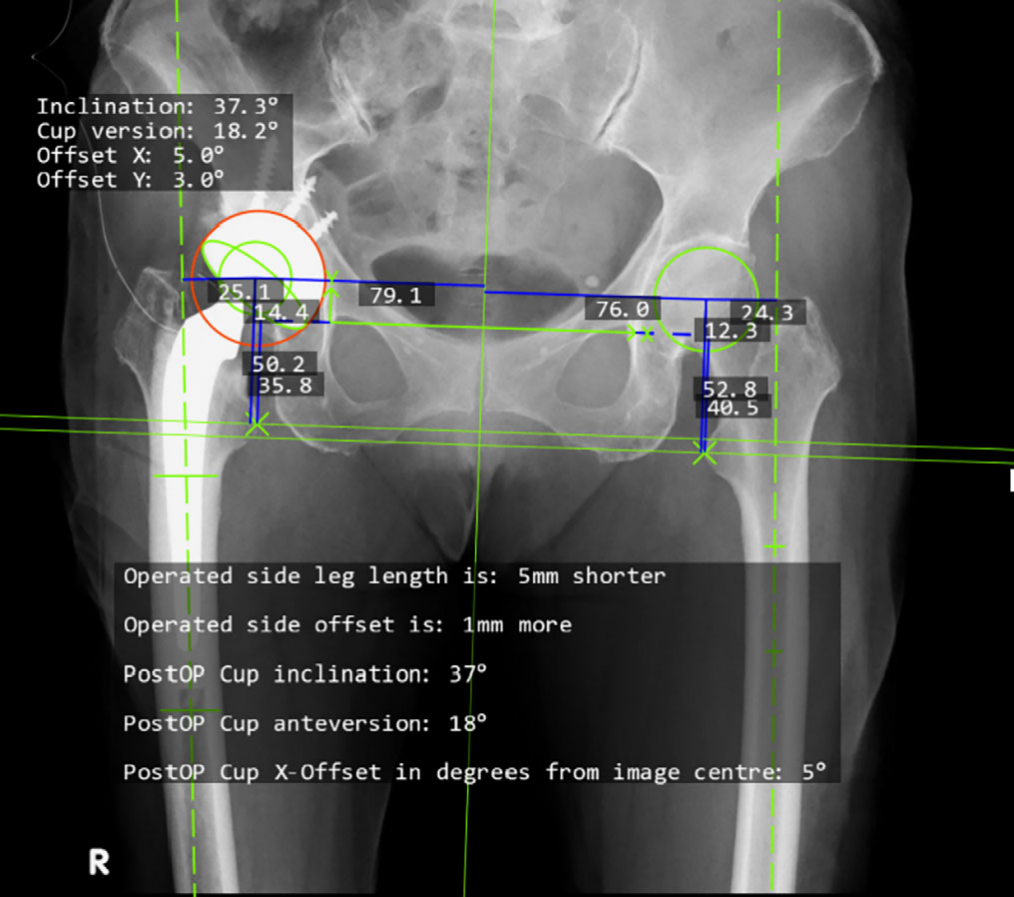
Measurement of parameters. Since the size of the patient's cup is known, data for that cup is entered into the system to obtain the true scale and actual distance for other parameters of the pelvis. The cup is outlined using the anteversion measurement tool, and the software automatically calculates the anteversion angle

Preoperative and postoperative radiographs of the anteroposterior (AP) pelvis were taken in all patients, and preoperative standing position radiographs were also obtained where possible. The imaging parameters, including the abduction and anteversion angle of the acetabular cup, were evaluated using the mdesk™ imaging measurement software (RSA Biomedical, Umea, Sweden) (Fig. 1). Leg length discrepancy (LLD) was measured using the inter‐teardrop line and the height of the lesser trochanter on anteroposterior hip views. PTA was calculated from the AP radiograph using Doiguchi *et al*.'s method.^13^ We measured the maximum transverse pelvic ring diameter (T) and the distance from the level of the pubic symphysis to the lower border of the sacroiliac joint perpendicular to the maximum transverse diameter (L) on the AP pelvic radiograph. Our calculations used the conversion formula: in females, PTA (°) = −69 × L/T + 63.6; in males, PTA (°) = −67 × L/T + 55.7. The reported mean PTA was 20°.^13^ Among all cases in our study, the PTA in the standing position could not be measured in 19 hips, including femoral neck fracture because the patients could not stand. In one hip, the inferior border of the sacroiliac joint was located caudal to the pubic symphysis in the standing position. The PTA in this condition would be more than 63.6° in females and 55.7° in males, which is not applicable.

### Clinical Analysis

Patients were clinically evaluated by questionnaires before surgery and at the final follow‐up. We used the Japanese Orthopaedic Association Hip‐Disease Evaluation Questionnaire (JHEQ, scored from 0 to 84, with 84 representing maximal mental health, pain relief, and activities of daily living).^14^, ^15^ The results of the visual analogue (VAS) scale of the JHEQ (scaled 0–100, with 0 marking complete satisfaction) were assessed separately.^16^, ^17^ Intraoperative and postoperative complications were recorded, including dislocations, periprosthetic fractures, prosthesis loosening, and infections.

### Statistical Analysis

Fundamental descriptive statistical analysis was used to describe the study population. Values were expressed as means or percentages, and measures were expressed as mean ± standard deviation (^−^x ± s). The Shapiro–Wilk test showed normal distributions of abduction angle, anteversion angle, supine and standing PTA, so we applied parametric tests: paired sample *t*‐test for intra‐group comparison and independent sample *t*‐test for inter‐group comparison. The preoperative and postoperative LLD, JHEQ scores, and VAS were not normally distributed, so nonparametric tests were used: the Wilcoxon test for intra‐group comparisons and the Mann–Whitney U test for inter‐group comparisons. Statistical significance was set at *P* < 0.05. Social Sciences software ver.18.0 (SPSS Inc., Chicago, IL, USA) was used for all statistical analyses.

## Results

### Radiographic Outcomes

**Fig. 2:**
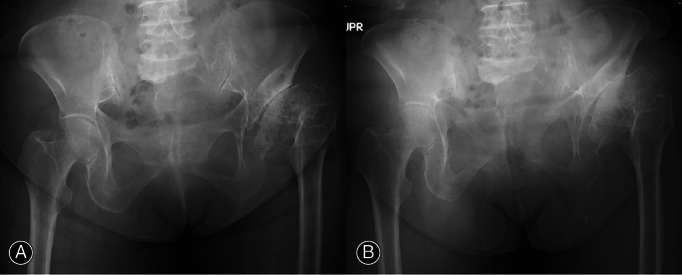
X‐ray of a patient with pelvic tilt and left hip osteoarthritis. Pelvic radiographs were taken in the standing and supine positions, and the pelvic tilt angle was calculated using the conversion formula described by Doiguchi *et al*.^13^ In this patient, the pelvic tilt angle was 48° in the supine position (A) and 55° in the standing position (B), with 7° of posterior pelvic tilt occurring during the postural transition

The mean PTA in patients in the supine position was 26.5° (8.6°–47.7°). The mean standing PTA in 78 patients was 34.5° (13.7°–58.2°) after excluding the 10 patients who could not be assessed and the 19 patients who could not stand. The difference in PTA between the standing and supine positions averaged 9.0° (−3.8°–25.0°). Sample radiographs in the supine and standing positions are shown in Figs Fig. 2A,B. In the supine position, the post‐operative abduction angle was 42.4° ± 5.8° (22.9°–53.8°), and the anteversion angle was 18.0° ± 5.3° (5.1°–32.8°). Regarding differences in limb length discrepancy, the mean preoperative LLD was −6.6 mm (−48 to 10 mm), and the mean postoperative LLD was 0.8 mm (−m to 19 mm). The lengthening of the lower limbs averaged 7.5 mm (−6mm to 27 mm). Patient pelvic and cup parameters are presented in Tables 2 and 3.

**TABLE 2:** Leg length discrepancy (LLD) and Japanese orthopaedic association hip disease evaluation questionnaire (JHEQ) scores

Parameter	Preoperative	Postoperative	*t* value	*P* value
LLD (mm)	−6.6 ± 9.2 (−48 to 10)	0.8 ± 7.2 (−25 to 19)	−10.66	*P* < 0.001
JHEQ total	22.4 ± 10.0 (2–44)	57.6 ± 17.3 (16–84)	−16.54	*P* < 0.001
Pain	7.3 ± 3.8 (0–20)	24.0 ± 5.0 (6–28)	−23.85	*P* < 0.001
ADL	5.6 ± 3.9 (0–15)	13.4 ± 8.2 (0–28)	−9.04	*P* < 0.001
Mental	9.6 ± 4.8 (0–23)	20.6 ± 7.3 (4–28)	−11.57	*P* < 0.001
VAS	83.5 ± 15.7 (30–100)	18.3 ± 27.3 (0–100)	15.74	*P* < 0.001

**TABLE 3:** Pelvic tilt angle and cup orientation

Parameter	Value
Pelvic tilt angle	
Supine position (*n* = 97)	26.5° (8.6°–47.7°)
Standing position (*n* = 78)	34.4° (13.7°–58.2°)
Cup acetabular abduction angle (supine position)	42.4 ± 5.8° (22.9°–53.8°)
Cup anteversion angle (supine position)	18.0 ± 5.3° (5.1°–32.8°)

### Clinical Outcomes

All patients had one year of routine follow‐up. The mean patient follow‐up was 22.2 months (11.5–50 months). The mean metal shell cup size was 50 mm (range: 46–56 mm), the mean polyethylene bearing liner diameter was 40 mm (36–44 mm), and the mean number of screws for fixation was three (1–4). The mean preoperative and final follow‐up JHEQ scores were 22.4 and 57.6, respectively (*P* < 0.001). The mean preoperative and final follow‐up VAS scores were 83.5 and 18.3, respectively (*P* < 0.001). Patient functional scores are presented in Table 2. There was one intraoperative complication with calcar fracture on the femoral side and no intraoperative complications on the acetabular side. Postoperative complications included early infection in one case (1.0%) and dislocation in one case (1.0%). Notably, the line‐to‐line and additional screw fixation techniques described in this study were associated with no instances of acetabular fracture or other complications on the acetabular side.

### Dislocation

The patient who experienced dislocation had an abduction angle of 50.2° and an anteversion angle of 20.9° at the primary THA. The supine PTA was 26.9°, the standing PTA was 39.8°, and the difference in PTA between supine and standing was 12°. One month postoperatively, dislocation occurred when the patient stood up from the floor and was reduced under fluoroscopy. Two months postoperatively, the dislocation occurred again under the same circumstances. Therefore, 2.5 months postoperatively, the patient underwent revision using a constrained support surface. Fifty months postoperatively, the patient underwent open revision and liner exchange after a failed closed reduction following a third dislocation.

## Discussion

In our cohort, the mean PTA was 26.5° in the supine position and 34.4° in the standing position, with a mean change in pelvic tilt of 9°. There was a significant improvement in postoperative LLD (0.8 *vs* −6.6 mm, *P* < 0.001), JHEQ score (57.6 *vs* 22.4), and VAS pain score (18.3 *vs* 83.5) compared to the preoperative, and only one patient experienced postoperative dislocation (1.0%). The imaging results and clinical outcomes were highly satisfactory.

### Challenges of Traditional “Safe Zones” and Pelvic Tilt

Despite a range of measures to prevent postoperative dislocation during THA surgery, such as adjusting the anteversion angle or selecting a high‐sided anti‐dislocation liner or a dual mobility cup system, hip dislocation remains a common complication after THA.

The “Lewinnek safe zone” for acetabular prostheses has long been associated with a low incidence of hip dislocation when the anteversion angle is within 15° ± 10°, and the abduction angle is within 40° ± 10°.^18^ However, some studies show that the patient's acetabular orientation is not static and suggest that more emphasis should be placed on the functional position of the acetabulum.^19^, ^20^ A change in the posterior pelvic tilt of more than 13° from a supine position to standing is defined as adverse pelvic mobility. Such movement can result in a 10° change in the acetabular anteversion angle, which may place the acetabular prosthesis outside the “safe zone” and cause postoperative dislocation.^21^, ^22^ Uemura *et al*.^23^ reported that the changes in pelvic sagittal inclination (PSI) from a supine to a standing position would exceed 10° in 8% to 19% of patients with end‐stage OA. Abdel *et al*.^24^ found that in the majority of cases where dislocation occurred, the cup was positioned in the safety zone, and he suggested that the traditional safety zone may no longer be applicable. In contrast, in our study, 21% (21/97) of the patients had a change in PTA of more than 13° during the position transition. Also, nine patients had an acetabular abduction angle, and six patients had an anteversion angle that was not within the “safe zone.”

### High Risk of Dislocation in Elderly Patients

The stability of hip replacement components is multifactorial, especially in patients who are elderly, with concomitant diseases, and at high risk for posterior pelvic tilt, as in this study. Fessy *et al*.^25^ have proposed that older age (>70 years for women and >75 years for men) is an independent factor that makes this age group relatively susceptible to complications of postoperative dislocation. Concomitant diseases can be another important cause of postoperative dislocation, and some of these etiologies are specific to patients of older age, such as: failed hip osteosynthesis, femoral neck fracture, cerebral palsy, Parkinson's, dementia, Alzheimer's, and American Society of Anesthesiologists (ASA) score >3. All patients in our study were more than 70 years old, and 89 had risk factors for dislocation (91.8%). However, only one patient in our cohort experienced postoperative dislocation. This outcome strongly suggests that DMC offered positive dislocation‐prevention effects following THA in our high‐risk cohort.

### Advantages of Dual Mobility Cup

The dual mobility cup system, designed by Bousquet *et al*.^26^ and originating in France in the 1970s, used a large head‐to‐neck ratio to increase stability and reduce prosthesis friction while increasing joint flexibility and reducing the risk of dislocation.^27^ Terrier *et al*.^28^ performed a finite element analysis of three types of prostheses and found that the DMC had a greater range of motion than other diameters (22mm, 32 mm) of femoral head prostheses. The dual mobility cup systems also compare favorably to other systems regarding the amount of von Mises stress within the polyethylene and the volume of polyethylene undergoing stress greater than 80% of the elastic limit.^28^ In a recent systematic review of 17,908 THAs with DMC implants, De Martino *et al*.^29^ demonstrated a mean dislocation rate of 0.9% in primary DMC‐THA and 3.0% in revision DMC‐THA. Cypres *et al*.^30^ showed a survival rate of 95.9% for the DMC and 99.1% for the femoral stem component after 12 years of follow‐up. Assi *et al*.^31^ used DMC to treat 125 patients with femoral neck fractures and found after a mean follow‐up of 39.6 months that 90% of patients described their surgery as a “forgotten hip.” No patients had experienced dislocation or loosening events. Prudhon *et al*.^32^ performed DMC‐THA in 105 patients with a mean age of 78. Follow‐up results showed a low dislocation rate (0.9%) and a 95% 10‐year expected survival rate.

### Strengths and Limitations

The strengths of our study are the adequate sample size and the considerable reliability of the results. At the same time, the relationship between the risk of postoperative dislocation and pelvic tilt in elderly patients with DMC‐THA has rarely been reported in the literature. Nevertheless, there are some limitations to our study. First, patient follow‐up was relatively short. However, the literature suggests that most dislocations occur in the early postoperative period.^2^, ^3^ Second, because of limited patient data, we were not able to obtain actual values for anteversion angles in the supine and standing positions, but only a qualitative analysis of the changes in the anteversion angles based on the geometric relationships between the PTA and the anteversion angle that were reported in previous studies. Finally, we used only one type of DMC, and our case series did not include a comparison with a standard cup. Additional direct techniques of measurement are needed in the future to obtain more precise pelvic data.

## Conclusion

In conclusion, even though all patients in this study were elderly, some had cup orientation outside the safe zone, and some had hypermobility of the pelvis, the dislocation rate was only 1%. This result suggests that DMC is a safe and effective treatment option for elderly patients at high risk of dislocation.

## Author Contributions

Mingliang Chen, Eiji Takahashi, Ayumi Kaneuji and Norio Kawahara designed the study. Eiji Takahashi and Yoshiyuki Tachi obtained the data. Makoto Fukui wrote the initial draft. Makoto Fukui and Yugo Orita performed the statistical analysis. Eiji Takahashi, Ayumi Kaneuji and Norio Kawahara reviewed and edited manuscripts. You Zhou, Eiji Takahashi and Toru Ichiseki assured the data and analysis were accurate.
